# Cigarette Smoking and Dating App Use: Findings from a Survey in a Sample of Adults in Italy

**DOI:** 10.3390/ejihpe11020040

**Published:** 2021-06-15

**Authors:** Luca Flesia, Valentina Fietta, Carlo Foresta, Merylin Monaro

**Affiliations:** 1 Unit of Andrology and Reproductive Medicine, Department of Medicine, University of Padova, 35128 Padova, Italy; carlo.foresta@unipd.it; 2 Department of General Psychology, University of Padova, 35131 Padova, Italy; valefietta.vf@gmail.com (V.F.); merylin.monaro@unipd.it (M.M.)

**Keywords:** cigarettes, geosocial networking smartphone applications, mobile dating apps, online dating, smoking, tobacco use

## Abstract

Existing studies in the literature indicate an association between the use of dating apps and substance-related behaviours (i.e., alcohol consumption, drug consumption). However, to date, no studies investigated the relation between dating app use and smoking. This study aims to explore this association. A total of 1278 respondents completed an online ad hoc questionnaire assessing demographics, smoking habits, dating app use, motivations for using dating apps. Multiple logistic regression analyses were run to investigate the relation between demographics and dating apps use on tobacco consumption. Being active user was significantly associated with being smoker, light daily and moderate-to-heavy smoker. Among users, using apps with the motive of searching for friends accounted for lower odds of smoking, light daily smoking and moderate-to-heavy smoking. However, heavy dating app users were less likely to smoke, to be light daily smokers and to be moderate-to-heavy smokers. The study indicates an association between using the apps and smoking, suggesting that motives for using the apps and intensity of use may moderate this association.

## 1. Introduction

Mobile dating apps (or “geosocial networking smartphone applications”) are applications that people can download on mobile phones, providing the opportunity to create new personal connections, usually with the goal of developing personal, romantic or sexual relationships. They allow users to find people based on different personal desired features (e.g., age, sex assigned at birth, sexual orientation) and, using featured geolocation systems, to find close located people. In the last decade, with the rise of smartphone use, dating apps have become very popular, profoundly changing the way people experience sexuality, romances and relationships [1]. Existing studies in the literature largely focused on investigating the impact of using smartphone dating applications on sexual risk behaviours, finding that dating app users are more prone to endorse high-risk sexual behaviours than non-users [2,3]. Some of them also focused on the association between dating app use and substance-related behaviors during sexual activities, more specifically investigating alcohol use and drugs consumption [4,5]. As regards the association between the use of dating apps and substance-related behaviours out of sexual activities, very few studies investigated this issue [6,7,8]. Holloway et al. (2015) found high rates of recent binge drinking, marijuana use and illicit substance use among a sample of 295 MSM app users [7]. Similarly, Winetrobe et al. found high levels of both binge drinking and drug use in the previous month among 146 young MSM Grindr users living in Los Angeles [6]. Phillips et al., investigating the association between drug consumption and dating app use among 379 American MSM, found higher odds of drug consumption among app-users, compared to non-users [8]. All of these studies regarded only MSM (men who have sex with men) app-users. Almost all lacked comparison groups from both heterosexual, female and non-users populations. Moreover, in this case also, they only considered alcohol consumption or drugs consumption.

Interestingly, to the best of our knowledge, no studies focused on the association between cigarette smoking and dating app use. However, there is no reason to exclude some possible association between them. Indeed, there is some evidence about significant associations between smoking and electronic media communication (EMC). According to the “stimulation hypothesis”, Valkenburg and Peter assumed that EMC may increase adolescents’ substance use influencing their time spent with friends: adolescents who use EMC to communicate with friends would also spend more face-to-face time with friends; as substance use typically takes place in contexts where young people socialize in person, EMC may be associated with more substance use [9]. Consistent with this, Gommans et al. found a positive association between EMC and substance use (tobacco, alcohol and cannabis): Dutch adolescents having higher levels of EMC were also more likely to smoke more cigarettes and consume more alcohol or cannabis; EMC explained unique substance use variance over and beyond face-to-face interactions [10]. Chiao et al. found that adolescent Internet use, particularly when it took place in Internet café, had a significant impact on future Taiwanese adolescents’ cigarette smoking and that the motive for Internet use was a predictor of future cigarette smoking. They also found that time spent using the Internet was associated with higher levels of risky behaviours [11].

The literature on problematic internet use (PIU) is also quite consistent in indicating a close positive and in some cases dose-dependent association between smoking and PIU [12,13,14]. PIU has been described in the literature as “internet addiction” or “pathological internet use”: it refers to addictive behavior and may include excessive or poorly controlled urges or behaviors regarding computer use and internet access that can lead to impairment or distress [15].

An initial exploratory approach to approach the specific topic of dating app use and tobacco consumption may be of advantage for many reasons. Although dating apps are one example of EMC, they are different from other electronic media use, like internet use or social media use (i.e., Facebook or WhatsApp). Dating apps are geolocated and specifically aimed at meeting new nearly located people. Therefore, the links between dating app use and smoking might be different compared to the links between other EMC and cigarette use. Investigating such specific topic may serve an initial step to open new lines of research. Moreover, tobacco consumption is extremely widespread and it is also one of the major global risk factors for disability and premature death [16,17]. Therefore, given the significant health effects and the public health effects of cigarettes smoking [16,17] and the spreading popularity of dating apps [18], such investigation might be very useful for giving cues to implement targeted and effective preventive programs.

The present study’s first aim is to investigate the possible differences in odds of cigarette smoking between active app users, former users and non-users, in order to investigate this association. Identifying such differences might open new research lines on the possible underlying mechanisms between dating app use and tobacco consumption.

Previous literature indicates that young adults and the MSM population are more prone both to use dating apps [2] and to endorse risk and unhealthy behaviours [19,20,21]. The present study’s second aim is to investigate the role of some demographics (e.g., sexual orientation, age, sex assigned at birth) on tobacco consumption.

Finally, the literature on dating apps highlighted that people can largely differ in their motives for using dating apps [22] and that such differences are associated with differences in behavioral patterns [23]. Moreover, a dose-dependent association between internet use and tobacco consumption was previously found [10,12]. The present study’s third aim was to investigate the relation between different patterns of dating app use (i.e., motives for use and intensity of use) and tobacco consumption. Differentiating app users according to their patterns of use may add context that would be beneficial for tailoring preventive interventions among app users to reduce health risks. It could also help to understand the associations between dating app use and cigarette smoking.

**Hypothesis** **1.** *Active app users, former users and non-users would differ for the odds of smoking: Active users would show higher odds of smoking than former users and non-users.*

**Hypothesis** **2.** *Age and sexual orientation would account for a portion of variance in the relation between dating app use and cigarette smoking.*

**Hypothesis** **3.** *The odds of smoking among active app users would differ according to motives for dating app use. The odds of smoking among active app users using intensively the apps would be higher than among non-users or former users.*

## 2. Materials and Methods

### 2.1. Participants and Procedure

This is a cross-sectional exploratory study. Data were collected between 1 June 2019 and 30 September 2019. The study was advertised on social media (e.g., Facebook and WhatsApp) and participants were recruited on a voluntary basis via an online link that directed them to the study survey. All participants provided their informed consent before starting the questionnaire. They received no compensation for participation. The procedure was clearly explained, and participants could quit the survey at any point without explaining their reasons. A total of 1390 respondents accessed the survey; 112 of them were excluded: 43 aged less than 18 years (legal age in Italy), 40 quitted the questionnaire before finishing it, 29 withdrew their informed consent. The final sample consisted of 1278 participants (age: M = 27.94, SD = 7.85; sex assigned at birth: males = 36.30%, females = 63.69%; years of education: M = 15.20, SD = 2.59). Specific demographic information of participants samples (active users, former users and non-users) is reported in Table 1.

The experimental procedure was designed in accordance with the Declaration of Helsinki. The project was approved by the Ethical Committee for the Psychological Research of the University of Padova (Prot. n. 3049).

### 2.2. Measures

The online questionnaire was developed ad hoc for this study. It consisted of 21 multiple-choice questions, assessing demographic information, dating app use and tobacco consumption (see Supplementary Materials for the questionnaire items):

Demographic information: participants were assessed for sex assigned at birth, age, educational level, relational status, and sexual orientation.

Tobacco consumption: participants were assessed for tobacco consumption (they were asked if they ever smoked cigarettes in the last 12 months). If yes, how much: “never or rarely (less than weekly)”; “weekly, up to 2 cigarettes per week”; “daily, up to 5 cigarettes a day”; “daily, from 6 to 10 cigarettes a day”; “daily, more than 10 cigarettes a day”. For subsequent analyses, participants were subdivided in 3 categories on the base of their tobacco consumption: smokers (weekly, up to 2 cigarettes per week), light daily smokers (up to 10 cigarettes a day) and moderate-to-heavy smokers (more than 10 cigarettes a day), according to previous studies [24,25,26].

Use of dating apps: participants were asked whether they were actually using (active users), had used but were no longer using (former users), or had never used any dating apps (non-users). Participants were informed that “dating apps” were intended as “online smartphone dating applications based on geosocial networking”. If they were former users, they were asked for duration of past use. Instead, active users were asked for: age at which they began using dating apps; number of years of apps use; number of dating apps installed).

Patterns of dating apps use (motives and intensity of use): active users were also assessed for primary aim when using dating apps (the following options were provided: “meet new people”, “have casual sex”, “begin a relationship”, “entertainment”, “I don’t know); response options are in line with those from the study by Fowler and Both (2020) [27]. Finally, active users were assessed for risk of problematic use of dating apps. They were asked about: number of accesses (“almost never”, “once or twice a month”, “once or twice a week”, “once a day”, “two or three times a day”, “more than three times a day”), frequency of notification check (“rarely without hearing the notification signal or vibration, or only hearing signals or vibrations”, “sometimes also without hearing the notification signal or vibration”, “often also without hearing the notification signal or vibration”, “very often also without hearing the notification signal or vibration”), frequency of stopping other activities to check dating apps (“never”, “rarely”, “sometimes”, “often”, “very often”), daily time spent on dating apps (“less than 5 min”, “from 5 to 15 min”, “from 15 min to half an hour”, “from half an hour to one hour”, “from one to three hours”, “from three to six hours”, “I’m not able to quantify it”), perception of uncontrolled use of dating apps (“Never”, “Rarely”, “Often”), frequency of unaware accesses to the apps (“Never”, “Rarely”, “Often”), frequency of giving up hours of sleep to check notifications (“Never”, “Rarely”, “Often”), desire to reduce the time spent in the apps (“Never”, “Rarely”, “Often”) and anxiety feelings if unable to use the apps (“Never”, “Rarely”, “Often”).

A brief description of each analysed variable is reported in Supplementary Materials. Data are available at the following repository: http://doi.org/10.5281/zenodo.4707318 (accessed on 1 June 2021).

### 2.3. Data Analysis

Analyses were computed using the open-source software JASP and R [28,29].

To investigate the association between two continuous variables the Pearson’s product-moment correlation (*r*) was computed. The independent sample *t*-test (*t*) was run to compare means of two independent groups; Cohen’s *d* was reported as a measure of effect-size (small *d* = 0.2, medium *d* = 0.5, and large *d* = 0.8). To investigate the association between two categorical variables the chi-squared (*χ*^2^) statistic was calculated, reporting the odds ratio (*OR*) or the standardized residuals (*z*) when results were significant (note that if *z* lies outside ±1.96 then it is significant at *p* < 0.05, if it lies outside ±2.58 then it is significant at *p* < 0.01, if it lies outside ±3.29 then it is significant at *p* < 0.001) [30]. The significance level of *p* value was set at 0.05, explicitly reporting when *p* was < 0.01 and <0.001.

Finally, multiple logistic regressions were run to investigate the association between a categorical dependent variable and multiple independent variables. The analysis was performed using the stepwise variable selection method, which identified predictors with a significant (*p* < 0.05) individual association with the outcome. It should be noted that the collinearity assumption was checked before running the model and it was not violated by any of the independent variables entered in the regression model (variance inflation factor (VIF) < 10 and tolerance was > 0.1) [31].

## 3. Results

### 3.1. Descriptive Statistics

On the entire sample, 43.43% of the participants declared they had been smoking at least once a week in the past 12 months. However, just 29.66% were light daily smokers. Finally, 7.75% of the participants were moderate-to-heavy smokers. In Table 2, the percentage of participants who declared to use tobacco is reported separately for each subsample (non-users, former users, active users). Demographic information of participant samples (active users, former users and non-users) is reported in Table 1.

### 3.2. Association between Use of Dating Apps and Frequency of Smoking (Hypothesis 1)

Investigating the role of dating apps, results showed a significant difference between non-users, active users and former users as regards smoking (*χ*^2^ = 15.25, *p* < 0.001). Indeed, the standardized residuals indicated that among the active users there were significantly more smokers than expected (*z* = 2.18). Comparing the active users and the other participants (non-users and former users), the odds of smokers were 1.56 times higher between the active users than other participants (*χ*^2^ = 10.86, *p* < 0.001). Similar results emerged analyzing the association between app use and light daily smoking (active users vs. former users vs. non-users: *χ*^2^ = 11.61, *p* < 0.01, standardized residuals of active users light daily smoking z = 2.05; active users vs. other users: *χ*^2^ = 7.68, *p* < 0.01, *OR* = 1.48). This association is even more evident for moderate-to-heavy smokers, as among active users there were significantly more moderate-to-heavy smokers than expected; moreover, among non-users there were significantly less moderate-to-heavy smokers than expected (active users vs. former users vs. non-users: *χ*^2^ = 16.72, *p* < 0.001, standardized residuals of active users who were moderate-to-heavy smokers z = 2.92, standardized residuals of non-users who were moderate-to-heavy smoker z = −2.55). Comparing active users and the other participants (non-users and former users), the odds of moderate-to-heavy smokers were 2.11 times higher between the active users than other participants (*χ*^2^ = 11.92, *p* < 0.001). Results about all the associations between type of dating apps users’ variable (active users vs. former users vs. non-users) and frequency of smoking are reported in Table S1 (see Supplementary Materials).

### 3.3. Role of Demographic Variables (Hypothesis 2)

Comparing males and females, the chi-squared analysis revealed that the odds of people smoking were lower in males than females (*χ*^2^ = 5.79, *p* < 0.05; *OR* = 0.75). However, the odds of moderate-to-heavy smokers were higher in males than females (*χ*^2^ = 5.79, *p* < 0.05; *OR* = 1.65). An association was found between sexual orientation and smoking (*χ*^2^ = 4.04, *p* < 0.05; *OR* = 0.79): in our sample the odds of people smoking is lower in people with heterosexual orientation than non-heterosexual. A negative correlation was found between age and smoking (*r_pb_* = −0.06, *p* < 0.05) and a positive correlation between age and moderate-to-heavy smoking (*r_pb_* = 0.06, *p* < 0.05). Finally, a statistically significant association was found between smoking and relational status (single vs. be involved in a relationship), (*χ*^2^ = 8.21, *p* < 0.01; *OR* = 1.39). Results about all the associations between demographic variables and frequencies of smoking are reported in Table S1.

As it emerged that smoking was associated with sex assigned at birth, sexual orientation, age, relational status and dating apps use, a multiple logistic regression analysis was run to investigate the role of each variable in predicting the variance of being a smoker. The dichotomous “being a smoker” variable was set as the dependent variable, while sex assigned at birth, sexual orientation, age, relational status, and dating apps use were entered as covariates. The final model accounted for a significant proportion of the variance of the dependent variable (McFadden R^2^ = 0.03, Nagelkerke R^2^ = 0.04, Tjur R^2^ = 0.01, Cox & Snell R^2^ = 0.04, *p* < 0.05, AUC = 0.61). This analysis highlighted that being an active user, being female, being younger and being single were predictors of smoking, while the sexual orientation did not account for a significant proportion of the variance, even if from the chi-squared analysis it emerged that non-heterosexual orientation was associated with smoking. Results are reported in Table 3.

A second logistic regression was run to predict moderate-to-heavy smoking, entering in the model the independent variables that resulted associated with it according to the previous Chi-squared analysis: sex assigned at birth, age and dating apps use. The final model accounted for a significant proportion of the variance of the “moderate-to-heavy smoking” dependent variable (McFadden R^2^ = 0.02, Nagelkerke R^2^ = 0.03, Tjur R^2^ = 0.01, Cox & Snell R^2^ = 0.01, *p* < 0.001, AUC = 0.61). This analysis highlighted that only dating apps use is a predictor of moderate-to-heavy smoking, specifically not being a non-user, while age and sex assigned at birth did not account for a significant proportion of the variance, even if the Chi-squared revealed that being male and older were associated with heavy smoking. Results are reported in Table 4.

It is worth noting that the multiple logistic regression for light daily smoking as a dependent variable was not computed as the dating app use turned out to be the only independent variable associated with light daily smoking (none of the demographic variables resulted to be significantly associated).

### 3.4. Role of Patterns of Dating App Use (Hypothesis 3)

Considering just the active users, we tested the differences between groups (smokers vs. no-smokers, light daily smokers vs. no-light daily smokers and moderate-to-heavy smokers vs. no-moderate-to-heavy smokers) in the intensity of dating apps usage. To address the problem of multiple testing, the Bonferroni correction was applied, dividing the *p*-value by the number of tested variables (=12) and setting the significance level at 0.004. Results are reported in Table 5. Statistically significant results emerged in the difference between smokers and no-smokers as concerns the frequencies in checking app notifications (*t* = 3.70, *p* < 0.004, *d* = 0.44) and in stopping other activities to check the apps (*t* = 3.54, *p* < 0.004, *d* = 0.42).

However, it is interesting to comment on the results even when the multiple testing correction is not applied. Indeed, a negative association emerged between both smoking and light daily smoking and higher frequencies in checking app notifications, giving up hours of sleep to check app notifications, having planned to reduce the time spent using the apps and getting anxious or missing something when they do not have the possibility of using the apps. The age at which active users began to use the apps was negatively associated with smoking and light daily smoking. Other negative associations were found between the amount of time per day spent in dating apps and smoking and between entering apps without realizing it and light daily smoking. Moreover, stopping other activities to check dating apps is negatively associated with all the three levels of smoking, while the using the apps more than you would like is associated with smoking and moderate-to-heavy smoking.

Regarding the associations between the motives for using the apps and the different levels of smoking, we found that the current aim of using dating apps to meet new people was associated with all the three levels of smoking (smoking: *χ*^2^ = 10.91, *p* < 0.001, *OR* = 0.41; light daily smoking: *χ*^2^ = 10.78, *p* < 0.01, *OR* = 0.37; moderate-to-heavy smoking (*χ*^2^ = 10.20, *p* < 0.01; *OR* = 0.13)). Moreover, people who had not a clear idea about why they used the apps were more likely to be moderate-to-heavy smokers (*χ*^2^ = 6.82, *p* < 0.01; *OR* = 2.71). Results are reported in Table 6. Note that to address the problem of multiple testing, the Bonferroni correction was applied, dividing the *p*-value by the number of tested variables (=5) and setting the significance level at 0.01.

Finally, considering just the former users, no statistically significant associations were found between the usage of dating apps for a long time (more than six months) and be a smoker (*χ*^2^ = 0.27, *p* = 0.61), light daily smoking (*χ*^2^ = 0.28, *p* = 0.59) or be a moderate-to-heavy daily smoker (*χ*^2^ = 1.31, *p* = 0.25).

## 4. Discussion

The present study has explored, for the first time, the relation between cigarette smoking and dating app use in the general population. The study examined differences in smoking habits between app users, non-users and former users. The role of demographic variables and motives for installing and using the apps was also analysed. The present study contributes to previous data regarding the association between EMC and smoking, providing new and specific information about the specific topic of the relation between dating app use and smoking. Such an initial approach will serve as a guide for the planning of future related lines of research.

The results highlighted an association between tobacco consumption and dating app use. Being an active user was significantly associated both with smoking, light daily smoking, and moderate-to-heavy smoking. Results are in line with previous literature regarding the association between EMC and tobacco use [10,11], and confirm our Hypothesis 1. This result may be interpreted according to the “stimulation hypothesis” by Peter and Valkenburg [32]; alternatively, it may suggest the presence of a possible underlying factor triggering both dating app use and smoking. Being a former app-user, independently from the duration of use of dating apps, was not associated with smoking.

As regards the role of demographics (Hypothesis 2), being female, being younger and being single, together with dating app use, predicted higher odds of cigarette smoking. However, as regards daily smoking, being an active user was the only predictor of tobacco consumption. Similarly, although moderate-to-heavy smoking was also associated with being male and being older, only dating app use accounted for increased odds of moderate-to-heavy smoking. Although heterosexual participants were less likely to smoke cigarettes, this association did not account for a significant amount of variance, suggesting that there was not a direct influence between sexual orientation and tobacco use. This finding does not support our second hypothesis, but aligns with recent studies indicating complex relations between sexual minority identities and cigarette smoking [33]. Our second hypothesis found only partial support: indeed, only younger age (together with being female and single) emerged as a significant predictor of being a smoker. Smokers cite stress reduction as their primary motive for smoking [34]. However, literature suggests that different patterns of smoking are linked with specific motives: more specifically, occasional smokers are more likely to report “positive reinforcement” motives (e.g., novelty seeking, reward dependence, and pleasurable sensations during early experimentation with smoking), while daily smokers to report “negative reinforcement” motives (e.g., craving, symptoms of nicotine withdrawal like anxiety, irritability, difficulty concentrating, restlessness, depressed mood) [35]. This is consistent with our finding regarding the significance of the younger age as a covariate only for the global sample of smokers, not for daily smokers nor moderate-to-heavy smokers: young people are generally more prone to seek for novelty and new experiences [36]. Overall, although the regression models show relatively low *R^2^* values, our results indicate that dating app usage is a significant predictor of tobacco consumption with respect to other demographic variables. These results open new line of research about possible common latent factors underlying this association.

Results regarding the association between intensity of dating app use and smoking unconfirm our Hypothesis 3. Indeed, differently from what previous literature on the association between PIU and smoking experience suggested [12,37,38], heavy dating app users were less likely to smoke and, as a tendency, to light daily smoke and in some cases to be moderate-to-heavy smokers. According to the “Uses and Gratifications” theory [39,40], people actively use media to satisfy some social/psychological need. These divergences in results may depend on unique features of dating apps, compared to other media: differently from the internet, dating apps are primary used as a tool for interpersonal relationships and provide facilities (i.e., GPS) for social connections with nearly located people. Therefore, dating app use can have a significant socio-relational value [1], buffering some psychological or emotional distress, otherwise leading a smoking person to smoke a cigarette; as an equivalent of cigarettes, the use of dating apps may compensate the role of tobacco smoking, as a way to cope with stress or to gain affect regulation. In this regard, several studies reported the effects of loneliness and relational motives on tobacco consumption [41,42,43,44,45,46,47]: people can smoke to fill the void left by social avoidance, viewing cigarettes as a friend and as having some of the same characteristics as social stimuli [48,49,50]. Research also documented a relation between intensity of smoking and social anxiety, and that this association is mediated by affiliative attachment motives [49]. Similarly, research on dating apps drawn from the “social skill model” of PIU [51] and from the concept of “preference for online social interaction” (POSI) [52] evidenced that social anxiety influenced negative outcomes in problematic use of dating applications serially through POSI and compulsive use only among those high in loneliness [53,54]. In this sense, dating app use might become a substituting activity, replacing the “self-medicating” use of smoking [55] for alleviating personal discomfort or compensating the need itself for “self-medication”.

As regards the role of motives for using dating apps, the odds of being smoker, light daily smoker and moderate-to-heavy smoker were lower among people who reported using the apps with the motive of meeting new people. These results may support the “loneliness hypothesis” as a common underlying factor between smoking and dating app use. Several studies showed the role of individual loneliness in the motives for dating app use [56,57,58]: people who use the apps searching for new people to meet may actively install them as a strategy to reduce social isolation or a way to cope with social anxiety. Therefore, dating app use and cigarette smoking might be parallel strategies to cope with feelings of loneliness or social anxiety. Considering the significant socio-relational value of dating apps [1], we can assume that, amid people using the apps to search for meet new people, actively using the apps buffers the emotional distress otherwise triggering to tobacco consumption. This is also consistent with our results concerning demographics and tobacco consumption: indeed, previous studies showed that loneliness is higher in women and in people without a partner [59]. On the contrary, people who had not a clear idea about why they currently used the apps were more prone to be moderate-to-heavy smokers. Alexithymia, that is a personal trait characterized by the inability to identify and describe emotions [60], may account for this result. Several studies indicate an association between alexithymia and smoking [61,62], suggesting that smoking may be an attempt to manage undifferentiated and unpleasant sensations created by alexithymia [63]. However, as the response options for motives for dating app use were limited and did not offer a “other” option for people to select, it is also possible that those who chose “I don’t know” simply found that the other response options didn’t capture their motives. The study findings on the role of motives supported our Hypothesis 3.

The study findings provide interesting cues for targeting and implementing more effective preventive programs: for instance, dating apps could promote adds or links to information regarding tobacco-related health risks, especially for females, singles or young dating app users.

### Limits and Future Perspectives

We acknowledge that this study has some limitations worth noting. First, the participants were recruited through an online link, posted and advertised on social media: the online advertising allows recruiting large samples and guarantees anonymity, but it may not guarantee about the sample representativeness and the quality of the data as regards the reliability of responses and the understanding of questions by participants. Second, the measure we used was a non-validated questionnaire, that was implemented ad hoc for the present research. Finally, the use of categorical questions has limited the possibility to run some sophisticated analysis (e.g., SEM).

In order to better understand the psychological mechanisms underlying the associations between smoking and dating app use, future studies could investigate the influence of common personality-based antecedents, for instance assessing the role of loneliness, social anxiety and of other possible mediating variables. Future researches could also explore potential links between dating app use, compulsive behaviors, addictive behaviors, and cigarette smoking.

## 5. Conclusions

To conclude, the present study approached for the first time the specific topic of the relation between tobacco consumption and dating app use, providing cues for the planning of future lines of research. The study highlights some patterns of association between smoking and dating app use, suggesting the presence of some common latent variable, possibly a psychological or social feature, that may induce both behaviors. Results also show the effect of motives for using and installing the apps and the role of the intensity of app use. Given the worldwide diffusion of smoking consumption, its significant impact in terms of people’s general health, and the ongoing wide spread of dating apps as dating tools, results from the present study provide interesting cues for targeting and implementing more effective preventive programs.

## Figures and Tables

**Table 1:** Demographic information for non-users, former users, active user and total sample. Percentages were rounded by excess from 0.05 up and by defect from 0.05 excluded down.

	Non-Users	Former Users	Active Users	Total Sample
N	598	393	287	1278
Age	Average = 26.35 (SD = 7.29)	Average = 27.70 (SD = 7.19)	Average = 31.60 (SD = 8.62)	Average = 27.94 (SD = 7.85)
Sex assigned at birth	Males = 21.24% Females = 78.76%	Males = 37.15% Females = 62.85%	Males = 66.55% Females = 33.45%	Males = 36.30% Females = 63.69%
Education level	8 years = 4.18% 13 years = 41.64% 16 years = 23.91% 18 years or more = 30.27%	8 years = 4.33% 13 years = 36.64% 16 years = 27.23% 18 years or more = 31.81%	8 years = 2.79% 13 years = 35.54% 16 years = 18.82% 18 years or more = 42.86%	8 years = 3.91% 13 years = 38.73% 16 years = 23.79% 18 years or more = 33.57%
Sexual orientation	Heterosexual = 85.12% Non-heterosexual = 14.88%	Heterosexual = 56.49% Non-heterosexual = 43.51%	Heterosexual = 34.15% Non-heterosexual = 65.85%	Heterosexual = 64.87% Non-heterosexual = 35.13%
Relational status	Relationship = 71.40% Single = 28.60%	Relationship = 63.36% Single = 36.64%	Relationship = 29.97% Single = 70.03%	Relationship = 59.62% Single = 40.37%

**Table 2:** Percentage of active users, former users and non-users who declared to be smokers, light daily smokers and moderate-to-heavy smokers. Percentages were rounded by excess from 0.05 up and by defect from 0.05 excluded down.

	Non-Users	Former Users	Active Users
N	598	393	287
Smokers	38.29%	45.04%	51.92%
Light daily smokers	25.42%	31.30%	36.24%
Moderate-to-heavy smokers	4.85%	8.65%	12.54%

**Table 3:** Output of logistic regression model entering sex assigned at birth, sexual orientation, age, relational status, dating apps use (non-user vs. former user vs. active user) as predictors for being a smoker.

				Wald Test			95% Confidence Interval	
	*B (Estimate)*	*SE*	*OR*	*Wald*	*df*	*p*	*Lower Bound*	*Upper Bound*
(Intercept)	0.46	0.26	1.58	3.21	1	0.07	−0.04	0.96
Dating apps use (non-user)	−0.38	0.14	0.69	7.68	1	**<0.01**	−0.64	−0.11
Dating apps use (active user)	0.49	0.17	1.63	7.93	1	**<0.01**	0.15	0.83
Sex assigned at birth (being male)	−0.29	0.18	0.75	2.72	1	0.10	−0.63	0.05
Age	−0.02	0.01	0.98	7.60	1	**<0.01**	−0.04	−0.01
Relational status (being single)	0.42	0.16	1.52	7.04	1	**<0.01**	0.11	0.73
Sex assigned at birth (being male) * Relational status (being single)	−0.55	0.25	0.58	4.87	1	**<0.05**	−1.04	−0.06

*Note. R^2^* = 0.03 (McFadden), 0.04 (Nagelkerke), 0.01 (Tjur), 0.04 (Cox & Snell). *Model Deviance =* 1699.75, *AIC =* 1713.75, *BIC =* 1749.82, *df* = 1271, *Δχ^2^ =* 4.87, *p **<*** **0.05**, *AUC =* 0.61. Previous steps’ statistics are reported in the Supplementary Materials.

**Table 4:** Output of logistic regression model entering sex assigned at birth, age, dating apps use (non-user vs. former user vs. active user) as predictors for being a moderate-to-heavy smoker.

				Wald Test			95% Confidence Interval	
	*B Estimate*	*SE*	*OR*	*Wald*	*df*	*p*	*Lower Bound*	*Upper Bound*
(Intercept)	−2.36	0.18	0.10	172.54	1	<0.001	−2.71	−2.01
Dating apps use (non-user)	−0.62	0.26	0.54	5.61	1	**<0.05**	−1.13	−0.11
Dating apps use (active user)	0.42	0.25	1.51	2.69	1	0.10	−0.08	0.91

*Note. R^2^* = 0.02 (McFadden), 0.03 (Nagelkerke), 0.01 (Tjur), 0.01 (Cox & Snell). *Model* *Deviance* = 680.24, *AIC* = 686.24, *BIC* = 701.70, *df* = 1275, *Δχ^2^* = 16.35, *p* **< 0.001**, *AUC =* 0.61.

**Table 5:** Independent sample *t*-test (*t*) testing the differences between groups (smokers vs. no-smokers, light daily smokers vs. no-light daily smokers and moderate-to-heavy smokers vs. no-moderate-to-heavy smokers) in the variables related to dating app use in the sample of active users. Degrees of freedom (*df*) of all the *t*-test are = 285. The effect size (Cohen’s *d*) is reported. Note that applying the Bonferroni correction, the significance level is set at 0.004.

App Use	Smokers vs. No-Smokers			Light Daily Smoking vs. No-Light Daily Smoking			Moderate-to-Heavy-Smoking vs. No-Moderate-to-Heavy Smoking		
	*t*	*p*	*d*	*t*	*p*	*d*	*t*	*p*	*d*
Age of beginning to apps usage	2.30	<0.05	0.27	2.78	<0.01	0.34	1.26	0.21	0.22
Number of years of apps usage	1.52	0.13	0.18	−0.03	0.98	−0.01	−0.62	0.54	−0.11
Number of dating apps installed	1.19	0.23	0.14	−0.20	0.84	−0.02	1.10	0.27	0.20
Amount of time per day spent in apps	2.21	<0.05	0.27	1.66	0.10	0.21	1.36	0.17	0.25
Make a great number of access	1.86	0.06	0.22	0.50	0.62	0.06	0.77	0.44	0.14
Stop other activities to check apps	3.54	**<0.001**	0.42	3.27	<0.01	0.40	2.40	<0.05	0.43
Check notifications	3.70	**<0.001**	0.44	2.25	<0.05	0.28	0.37	0.71	0.07
Use the apps more than you would like	2.31	<0.05	0.27	1.36	0.17	0.17	2.31	<0.05	0.41
Access the apps without realizing it	1.50	0.14	0.18	2.03	<0.05	0.25	1.40	0.16	0.25
Give up hours of sleep to check app notifications	3.17	<0.01	0.38	2.33	<0.05	0.29	0.71	0.48	0.13
Have planned to reduce time in the apps	2.24	<0.05	0.27	2.05	<0.05	0.25	1.31	0.19	0.23
Get anxious or miss something without the possibility of using the apps	2.32	<0.05	0.27	3.12	<0.01	0.38	1.61	0.11	0.29

**Table 6:** Association between different levels of smoking and app usage current motivation in the sample of active users. Note that applying the Bonferroni correction, the significance level is set at 0.01.

	Smoking			Light Daily Smoking			Moderate-to-Heavy Smoking		
App Usage Motivation	*χ* ^2^	*df*	*p*	*χ* ^2^	*df*	*p*	*χ* ^2^	*df*	*p*
Entertainment	0.02	1	0.89	1.15	1	0.28	1.32	1	0.25
Meet new people	10.91	1	**<0.001**	10.78	1	**<0.01**	10.20	1	**<0.01**
Begin a relationship	3.44	1	0.06	0.55	1	0.46	3.56	1	0.06
Casual sex	0.68	1	0.41	0.29	1	0.59	1.52	1	0.22
I Don’t know	1.95	1	0.16	4.39	1	**<0.05**	6.82	1	**<0.01**

## Data Availability

Data available at the following repository: http://doi.org/10.5281/zenodo.4707318 (accessed on 1 June 2021).

## Supplementary Materials

The following are available online at https://www.mdpi.com/article/10.3390/ejihpe11020040/s1. Table S1: association between different levels of smoking and demographic variables and associations between different levels of smoking and app usage’s variables; Intermediate steps of the logistic regression reported in Table 3 of the main text; Questionnaire items; List of variables.
