# Supplementary material for: Cigarette Smoking and Dating App Use: Findings from a Survey in a Sample of Adults in Italy
Source: Eur J Investig Health Psychol Educ. 2021 Jun 15;11(2):557–69. doi: 10.3390/ejihpe11020040 (PMC8314363; doi:10.3390/ejihpe11020040)
Supplement: Supplementary file 1 [file ejihpe-11-00040-s001.zip › ejihpe-1212189-SUPPLr3.pdf]

# Cigarette smoking and dating app use: findings from a survey in a sample of adults in Italy

# Supplementary Materials

## Supplemental tables

**Table S1**: association between different levels of smoking and demographic variables and associations between different levels of smoking and app usage's variables.

| | Smoking | | | Light daily smoking | | | Moderate-to-heavy smoking | | |
|---|---|---|---|---|---|---|---|---|---|
| | $r_{pb}$ | | *p* | $r_{pb}$ | | *p* | $r_{pb}$ | | *p* |
| Age | -0.06 | | **<0.05** | -0.02 | | 0.51 | 0.06 | | **<0.05** |
| Educational level | -0.01 | | 0.73 | -0.02 | | 0.56 | -0.01 | | 0.64 |
| | $\chi^2$ | *df* | *p* | $\chi^2$ | *df* | *p* | $\chi^2$ | *df* | *p* |
| Being male | 5.79 | 1 | **<0.05** | 0.34 | 1 | 0.56 | 5.79 | 1 | **<0.05** |
| Being heterosexual | 4.04 | 1 | **<0.05** | 3.63 | 1 | 0.06 | 1.86 | 1 | 0.17 |
| Being single | 8.21 | 1 | **<0.01** | 3.50 | 1 | 0.06 | 0.42 | 1 | 0.52 |
| User subsamples (active vs former vs non-user) | 15.25 | 2 | **<0.001*** | 11.61 | 2 | **<0.01*** | 16.72 | 2 | **<0.001*** |
| Being an active user | 10.86 | 1 | **<0.001** | 7.68 | 1 | **<0.01** | 11.92 | 1 | **<0.001** |

*Note. OR:* 0.75 (smoking and being male), 1.65 (moderate-to-heavy smoking and being male), 0.79 (smoking and being heterosexual), 1.39 (smoking and being single), 1.56 (smoking and being an active user), 1.48 (light daily smoking and being an active user), 2.11 (moderate-to-heavy smoking and being an active user).

**Standardized residuals:* z = 2.18 (smoking and being an active user), z = 2.05 (light daily smoking and being an active user), z = 2.92 (moderate-to-heavy smoking and being an active user), z = -2.55 (moderate-to-heavy smoking and being a non-user),

## Intermediate steps of the logistic regression reported in Table 3 of the main text

Step 1 (dating apps use): *df*=1275, $\Delta\chi^2$=15.23, *p*<0.001 , $R^2$ = 0.01 (McFadden), 0.01 (Nagelkerke), 0.02 (Tjur), 0.01 (Cox & Snell); step 2 (dating apps use +sex assigned at birth): *df*=1274, $\Delta\chi^2$=17.28, *p*<0.001, $R^2$ = 0.02 (McFadden), 0.03 (Nagelkerke), 0.07 (Tjur), 0.03 (Cox & Snell); step 3 (dating apps use + sex assigned at birth + age): *df*=1273, $\Delta\chi^2$=9.55, *p*<0.01, $R^2$ = 0.02 (McFadden), 0.03 (Nagelkerke), 0.04 (Tjur), 0.03 (Cox & Snell); step 4 (dating apps use + sex assigned at birth + age + relational status): *df*=1272, $\Delta\chi^2$=2.85, *p*=0.09, $R^2$ = 0.03 (McFadden), 0.04 (Nagelkerke), 0.03 (Tjur), 0.04 (Cox & Snell).

## Questionnaire items

Indicate your sex assigned at birth:

- ◻ Male
- ◻ Female

Indicate your age:

______

Indicate your educational level (indicate the highest qualification obtained):

- ◻ Elementary school
- ◻ Secondary school
- ◻ High school
- ◻ Three-year degree
- ◻ Master's degree
- ◻ Master/Doctorate/Specialization

Indicate your relational status:

- ◻ Single
- ◻ In a relationship (stable or informal)

Indicate your sexual orientation:

- ◻ Heterosexual
- ◻ Homosexual
- ◻ Bisexual
- ◻ Fluid
- ◻ Other

Have you smoked cigarettes in the past 12 months? If so, how much?

- ◻ Never or rarely (less than weekly)
- ◻ Weekly, up to 2 cigarettes per week
- ◻ Daily, up to 5 cigarettes a day
- ◻ Daily, from 6 to 10 cigarettes a day
- ◻ Daily, more than 10 cigarettes a day

Do you use dating apps?

- ◻ No, I've never used them
- ◻ No, I used them in the past

- ◻ Yes, I do

Question for former users only

How much time have you used them?

- ◻ 1-5 days
- ◻ 6-30 days
- ◻ 1-6 months
- ◻ more than 6 months

Questions for active users only

Indicate the number of dating apps you have installed:

________

How many years have you been using apps?

________

At what age did you use a dating app for the first time?

________

How often do you enter apps?

- ◻ Almost never
- ◻ Once or twice a month
- ◻ Once or twice a week
- ◻ Once a day
- ◻ Two or three times per day
- ◻ More than three times per day

How often do you check your apps notification?

- ◻ rarely without hearing the notification signal or vibration, or only hearing signals or vibrations
- ◻ sometimes also without hearing the notification signal or vibration
- ◻ often also without hearing the notification signal or vibration
- ◻ very often also without hearing the notification signal or vibration

Do you ever stop what you're doing to check if you have app notifications?

- ◻ Never
- ◻ Rarely

- ◻ Sometimes
- ◻ Often
- ◻ Very often

On average, how much time do you spend in the apps in a day?

- ◻ less than 5 minutes
- ◻ from 5 to 15 minutes
- ◻ from 15 minutes to half an hour
- ◻ from half an hour to one hour
- ◻ from one to three hours
- ◻ from three to six hours
- ◻ I'm not able to quantify it

What is your current primary aim when using dating apps?

- ◻ Casual sex
- ◻ Begin a relationship
- ◻ Meet new people
- ◻ Entertainment
- ◻ I don't know

Do you happen to use the apps more than you would like?

- ◻ Never
- ◻ Rarely
- ◻ Often

Do you happen to access the apps without thinking about it and realize it later?

- ◻ Never
- ◻ Rarely
- ◻ Often

Do you happen to give up hours of sleep to check app notifications?

- ◻ Never
- ◻ Rarely
- ◻ Often

Have you ever proposed yourself to reduce the time you spend in the apps?

- ◻ Never
- ◻ Rarely
- ◻ Often

Do you get anxious or do you miss something if you can't use the apps for some reason?

- ☐ Never
- ☐ Rarely
- ☐ Often

## List of variables

The list reports the 29 variables that were extracted from the responses to the questionnaire.

**Being_male =** this is a dichotomous variable indicating the sex assigned at birth of the participant (1=male, 0= female).

**Age =** this is a continuous variable indicating the age of the participant.

**Education_level =** this variable indicates years of education of the participant. The response was coded as follows: 8= "middle school", 13= "high school", 16= "triennial degree", 18= "master degree or more years of education".

**Relational_status =** this is a dichotomous variable indicating the relational status of the participant (1=single, 0= in a relationship).

**Sexual_orientation =** this is a dichotomous variable indicating the sexual orientation of the participant (1= heterosexual, 0= non-heterosexual).

**Being_smoker** = this is a dichotomous variable indicating participants who smoked at least once a week in the past 12 months (1= smoking, 0= no smoking).

**Being_daily_smoker** = this is a dichotomous variable indicating participants who light smoke daily in the past 12 months (1= smoking daily, 0= minor frequency of smoking).

**Being_heavy_smoker** = this is a dichotomous variable indicating participants who smoke moderately-to-heavily (more than 10 cigarettes a day) in the past 12 months, (1= smoking heavily, 0= minor frequency of smoking).

**Dating_app_user =** this categorical variable indicates types of dating app users (former user, active user, non-user).

**Active_user =** this dichotomous variable indicates active dating app users (1= active users, 0 = other users).

**Beginning_age =** this is a continuous variable indicating the age at which active users installed dating apps.

**Usage_years =** this is a continuous variable indicating active users' years of dating apps utilization.

**Past_time =** this variable indicates how much time former users have used dating apps. The response was coded as follows: 1= "1-5 days", 2= "6-30 days", 3= "1-6 months" 4= "more than 6 months".

**Past_time_6_months =** this dichotomous variable indicates former dating app users who utilized for more than 6 months dating apps (1= more than 6 months, 0 = other utilization time).

**App_number =** this is a continuous variable indicating how many dating apps active users have.

**App_access =** this variable indicates how many times active users enter their dating apps. The response was coded as follows: 1= "almost never", 2 = "once or twice per month", 3 = "once or twice a week", 4 = "only once a day", 5 = "two or three times a day", 6 = "more than 3 times per day",

**Check_notifications =** this variable indicates how often per day active users check their dating apps notifications. The response was coded as follows: 1= "rarely check notifications without hearing the notification signal or vibration, or check notifications only hearing signals or vibrations", 2= "sometimes check notifications also without hearing the notification signal or vibration", 3= "often check notifications also without hearing the notification signal or vibration", 4 = "very often check notifications also without hearing the notification signal or vibration".

**Stop_activities =** this variable indicates how often active users stop other activities to check their dating apps. The response was coded as follows: 0= "never stop other activities", 1= "rarely stop other activities", 2= "sometimes stop other activities", 3= "often stop other activities", 4= "very often stop other activities".

**Daily_time =** this variable indicates how much time per day active users spend using dating apps. The response was coded as follows: 1= "less than 5 minutes", 2= "from 5 to 15 minutes", 3= "from 15 minutes to half an hour", 4= "from half an hour to one hour", 5= "from one to three hours", 6= "from three to six hours". Note: "not able to quantify it" responses were not included in the analysis.

**Uncontrolled_use** = this variable indicates how much the active users think they are using too much the apps. The response was coded as follows: 0 = "never", 1= "rarely", 2= "often".

**Unaware_accesses** = this variable indicates how much the active users enter dating apps without thinking of it and realizing it later. The response was coded as follows: 0 = "never", 1= "rarely", 2= "often".

**Give_up_sleep** = this variable indicates how much the active users give up hours of sleep to check app notifications. The response was coded as follows: 0 = "never", 1= "rarely", 2= "often".

**Desire_to_reduce_time** = this variable indicates how much the active users have proposed to themselves reduce the time spent on dating apps. The response was coded as follows: 0 = "never", 1= "rarely", 2= "often".

**Anxiety_feelings** = this variable indicates how much the active users get anxious or miss something when they are unable to use the apps. The response was coded as follows: 0 = “never”, 1= “rarely”, 2= “often”.

**Usage_entertainment =** this is a dichotomous variable indicating active users who currently use dating apps as a entertainment (1= as entertainment, 0 = other usages).

**Usage_new_people=** this is a dichotomous variable indicating active users who currently use dating apps to find friends (1= to find friends, 0 = other usages).

**Usage_begin_relationship=** this is a dichotomous variable indicating active users who currently use dating apps to find romantic partners (1= to find romantic partners, 0 = other usages).

**Usage_casual_sex =** this is a dichotomous variable indicating active users who currently use dating apps to find sexual partners (1= to find sexual partners, 0 = other usages).

**Usage_don't_know =** this is a dichotomous variable indicating active users who currently don’t know why there are using dating apps (1= no specific aim, 0 = other usages).
